# Prediction of Lower Third Molar Eruption in Panoramic Radiography Using Artificial Intelligence (AI): PDApp

**DOI:** 10.3390/diagnostics16040516

**Published:** 2026-02-09

**Authors:** Susana Santeiro-Hermida, Manuel Fernández-Delgado, Eva Cernadas, Paz Otero-Casal, Mercedes Gallas-Torreira

**Affiliations:** 1 Digital Dentistry Unit, Stomatology Area, Department of Surgery and Medical-Surgery Specialities, School of Dentistry, Faculty of Medicine and Odontology, Universidade de Santiago de Compostela, Rúa Entrerríos s/n, 15782 Santiago de Compostela, Spain; susana.santeiro@rai.usc.es (S.S.-H.); mariadelapaz.otero.casal@usc.es (P.O.-C.); 2 Centro Singular de Investigación en Tecnoloxías Intelixentes da USC (CiTIUS), Universidade de Santiago de Compostela, Rúa Xenaro de la Fuente Domínguez, 15782 Santiago de Compostela, Spain; manuel.fernandez.delgado@usc.es (M.F.-D.); eva.cernadas@usc.es (E.C.)

**Keywords:** artificial intelligence, oral radiology, third molar, panoramic radiography, machine learning, software

## Abstract

**Background/Objective**: Preoperative prediction of third molar eruption with artificial intelligence (AI) has been a challenge in dentistry and oral surgery. **Methods**: In this investigation, we used machine learning (ML) algorithms to characterize M3 panoramic radiological images for the preoperative differential diagnosis of third nolar eruption/retention and compared them with clibical explorations to validate their performance. **Results**: We retrospectively collected data of patients with mandibular thid molar retention, where all eruption diagnoses were confirmed via clinical exploration. A total of 383 panoramic radiographies were selected to train the PDApp software for eruption diagnosis for the software validation. **Conclusions**: The PDApp software achieved the highest performance metrics for the prediction of mandibular third molar eruption

## 1. Introduction

The decision to extract or retain lower third molars is usually made at the beginning or end of dental treatment planning by the clinician based on his or her clinical judgment and radiological follow-up, and it is not exempt from controversy. Between 50% and 80% of the global population is estimated to have at least one impacted tooth during their lifetime, and routine oral and radiological examinations are critical for early detection and management, helping to mitigate potential complications [1,2]. The use of computer-aided diagnosis based on deep learning is emerging in the field of dentistry with the advancement of artificial intelligence (AI) technology [3,4,5,6]. The accurate prediction of the eruption/noneruption (retention) status would allow the clinician to perform a timely extraction/exodontia before the retained position is acquired, which might increase intra- or postoperative complications. The panoramic radiograph is a widely used X-ray imaging technique for the diagnosis of impacted teeth and is the most commonly employed modality for their follow-up and for evaluating overall oral health [7].

Different radiographic techniques using two-dimensional measurements (panoramic or lateral radiographs) to assess the space for third molar eruption have been documented in the literature [6,7,8,9,10]. However, panoramic radiography yields the most accurate estimation in this field and is the most popular dental record for clinicians [6]. Regarding impacted lower third molars, determining the retromolar space and the third molar mesiodistal diameter and angulation/inclination contributes to earlier M3 eruption prognosis. According to Hattab and Alhaija [11], the most significant variable associated with M3 retention is the lack of space for eruption in the retromolar area. The authors measured the distance between the tangent to the distal surface of the lower second molar and the anterior edge of the ramus of the mandible, as well as the mesiodistal diameter of the lower third molar (the greatest distance between the mesial and distal surfaces of the M3 crown). Even with a large enough retromolar space, the eruption of the lower third molar cannot be guaranteed because other factors influence the skeletal growth pattern, the direction of the dentition eruption, the dental extractions performed, the morphology of the root, and the third molar maturation stage. It has been reported that the retromolar space and M3 mesiodistal angulation are the most important factors for M3 eruption [12,13].

The aim of the abovementioned studies and ours is the determination of a reliable and efficient predictive method. AI applications save time in treatment planning in implantology, orthodontics, and orthognathic surgery via the automated detection and segmentation of 3D images [3,4,5,6]. In this context, we developed and validated the Panoramic Dental Application tool (hereinafter termed the PDApp) for the prediction of M3 eruption potential in adolescent patients via linear and angular measurements [14], and we evaluated its accuracy in the prediction of lower third molar eruption/retention on panoramic radiographs.

## 2. Materials and Methods

**Figure 1:**
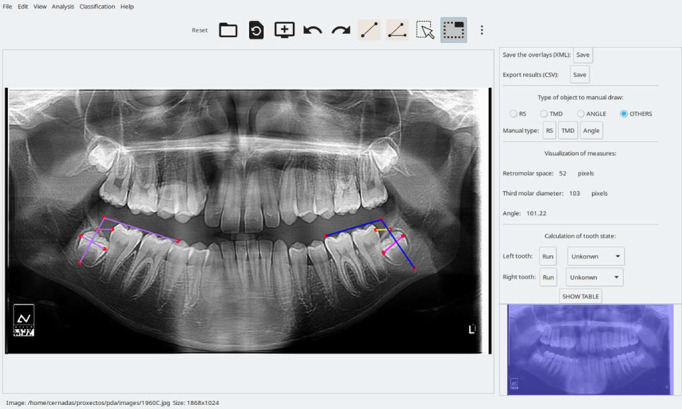
Screenshot of the PDApp software with the panoramic radiography analysis and lateral panel opened. In the left molar, the pink line represents the third molar diameter, the yellow line represents the retromolar space, and the blue lines represent the angle. The measures of the right molar are shown in the lateral panel.

The PDApp (v.1) is a desktop application that runs on a general-purpose computer under the Linux and Windows operating systems, and it was written in the C++ programming language using the GTK+ 3.24 (GIMP Tool Kit) library to develop the GUI. Figure 1 shows the GUI of the PDApp for the loading, processing, and reviewing of a typical radiological image by an expert and the lateral panel displayed.

The analyzed panoramic radiographies were retrospectively selected from a private radiological center (Centro de Radiología Príncipe, Vigo, Spain). We retrospectively collected the data of 35,415 patients with mandibular third molar retention, with their consent, and we anonymized all image data prior to analysis. In total, 383 panoramic radiographies were included: 94 for training and technical validation and 289 for posterior clinical validation. This study received approval from the Santiago-Lugo Research Ethics Committee, with registration code 2017/121. Radiographies were acquired with a CS 8100 device SN EBXG197 (Carestream Health Inc., Noisy-le-Grand, France).

The PDApp is a modular and extensible application composed of three layers: (1) the GUI layer, with which the user interacts through edition tools, including modules to draw and manage objects, set preferences, and interact with the software; (2) the logic application layer, which contains modules to measure distances and angles, predict the third molar potential, train the classifier, and calculate the statistical results; (3) the persistence layer, which stores all the data needed and calculated by the software, including modules to save the overlays on the image and the statistical results. The image overlays, which contain the analysis supervised by the experts, are stored in the popular text format XML (Extensible Markup Language), and the statistical results, calculated from the overlays, are stored in the known text format CSV (Comma-Separated Values), which is portable and can be imported from other spreadsheet software for further use.

**Figure 2:**
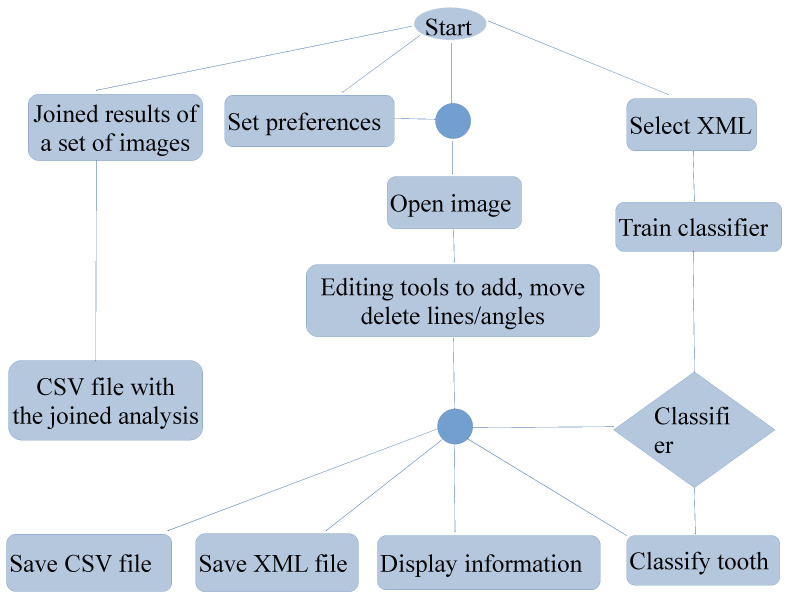
Flowchart of PDApp functionalities.

Figure 2 shows a flowchart with the main PDApp functionalities, which are accessible from the GUI. A typical working session for a user includes the following actions: (1) opening a radiological image; (2) manually drawing the retromolar space, third molar diameter, and angle for each third molar; (3) automatically measuring the distances and angles; (4) automatically classifying the third molar; (5) going to expert’s supervision, as described below; (6) saving the overlays drawn on the image into the XML file; (7) exporting the statistical measures to the CSV file; (8) the performance of any of the following optional functionalities at any time: setting preferences, saving the joined results of a set of patients, and training the classifier.

As mentioned in our previous study [14], the machine learning model included in PDApp is a support vector machine (SVM) with a radial basis function kernel trained with 188 molars cases distributed into 115 erupted (representing 61.2% of the data) and 73 retained (38.8%) molars. We used the LibSVM implementation in the C++ programming language, with kernel spread γ = 0.00012. The model uses two features, third molar angulation, and Radiological Retention Coefficient (RRC), both extracted from the panoramic radiological image. See the details of the training process in publication [15]. This trained SVM was evaluated on 539 third molars cases belonging to 289 other patients, achieving an accuracy of 97.96% in distinguishing between erupted and retained third molar. This high performance is the main strength of this model, although it is limited by the low number of training and test patterns which is caused by the difficulty to obtain clinical diagnosis data.

## 3. Results

The prediction of third molar eruption is a topic of great interest for clinicians (oral surgeons, orthodontists, and general dentists), as the eruption date can improve patient care. In the present work, we used the semi-automated AI tool PDApp for predicting the third molar eruption potential, which was technically and clinically validated in a prior study [14]. Specifically, we examined the ability of the PDApp tool to classify erupted and nonerupted/retained molars in other populations. For this purpose, we considered a new dataset composed by 148 patients, 98 retained (66.2%) and 50 erupted (33.8%), divided into the following groups: the RSO group, formed by patients with retained M3s who had not undergone orthodontic procedures (*n* = 47 M3s); the RCO group, formed by patients with retained M3s who had undergone orthodontic procedures (*n* = 51 M3s); and the E group, formed by patients with erupted M3s (*n* = 50 M3s). For this sample, the PDApp tool correctly classified 100% of the M3s studied.

**Figure 3:**
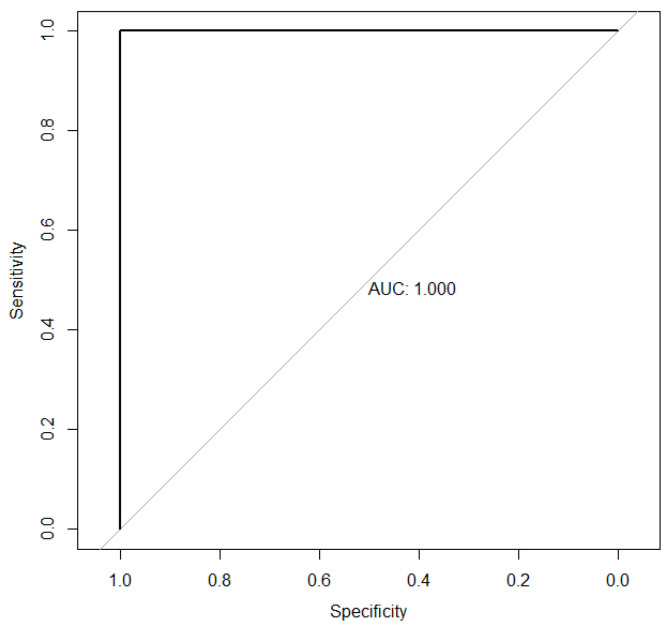
ROC curve associated with RRC (the gray and black lines define the random classification and classification using RRC variable respectively).

**Figure 4:**
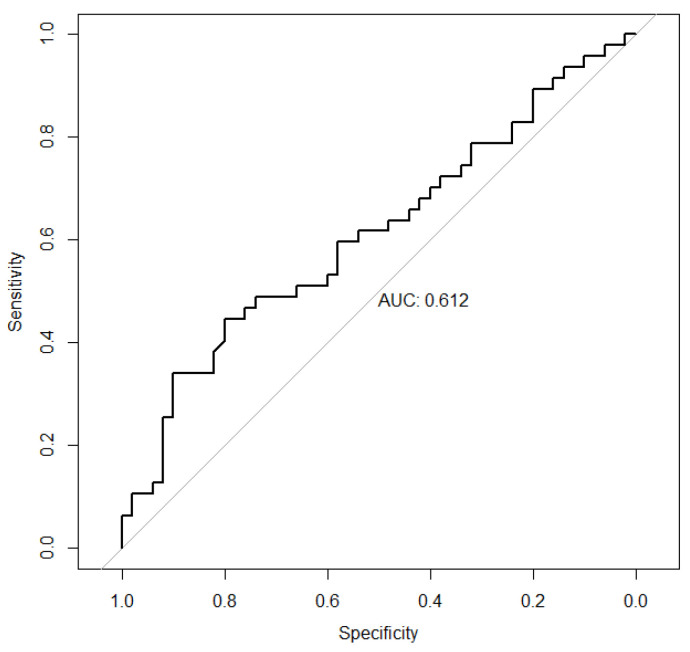
ROC curve associated with Cos erupθ.

Furthermore, we evaluated the ability of each single feature used by PDApp (radiological retention coefficient, RRC, and cosine of the eruption angle, Cos erupθ) to classify erupted and retained M3s. For this purpose, we fitted the ROC curves [15,16] showed in Figure 3 and Figure 4. For the RRC variable (Figure 3), the area under the associated curve is 1 (the maximum value reflecting perfect classification), highlighting its extremely high discriminatory power between the eruption and retention statuses. Additionally, the points of maximum sensitivity and specificity were reached, with values of 1 in both cases. The cutoff point where maximum sensitivity and specificity are achieved is 0.722. That is, M3s with retention coefficients lower than 0.722 are classified as “retained”, while those with retention coefficients higher than 0.722 are classified as “erupted”.

For the cosine of the eruption angle variable (Figure 4), the associated ROC curve is very close to the diagonal of the first quadrant. Thus, the area under the associated curve is 0.612, with a 95% confidence interval (CI) of (0.4986–0.7257) for this value. These results illustrate the low discriminatory power of the considered variable. Furthermore, the points of maximum sensitivity and specificity were reached, with values of 0.446 (95% CI: 0.234–0.5957) and 0.80 (95% CI: 0.50–0.927), respectively.

Subsequently, a descriptive analysis of the three study groups was performed. Table 1 shows the main characteristic measures associated with the RRC and the cosine of the eruption angle in the RSO, E, and RCO groups.

The retention coefficient in group E exhibits clearly different behavior from that of the retention coefficients in the RSO and RCO groups. For example, while the dispersion range of the retention coefficient in group E is 0.7313–2.2047, those of groups RSO and RCO are 0.1620–0.7135 and 0.1352–0.7109, respectively. That is, there is no overlap between the results of group E and those of groups RSO and RCO. Similarly, the central tendency measures, the means and medians, were as follows: RSO group: 0.3939 and 0.3845, respectively; RCO group: 0.3251 and 0.3032, respectively; and E group: 1.3319 and 1.2835, respectively. Analogous results were drawn from the calculation of the first or third quartile.

The characteristic measurements associated with the RRC in groups RSO and RCO are very similar to each other and very different from those in group E.

Based on the findings in Table 2, with a significance level of 5%, there is statistically significant evidence that the behavior of the RRC in group E is not the same as that in the RSO and RCO groups. Moreover, evidence allows us to state that the behavior of the RRC in the RSO and RCO groups is different.

From these results, it seems that we could design a rule-based system that classifies patients into third molar erupted and retained categories using only the RRC feature as input data. This system would classify patients with RRC ≤ 0.722 as retained, and patients with RRC > 0.722 as erupted. We performed an external validation of this rule-based classifier on the data used in [14] to train the SVM of PDApp (188 cases, 73 retained and 115 erupted). The confusion matrix achieved by this classifier is shown in Table 3, yielding an accuracy of 51.1%, with a sensitivity of 100%, specificity of 20%, F1-score of 61.3% and AUC of 81.5%. This result is fairly low, showing that the rule-based system is not robust to new data. This might be caused by differences between these new data and the data used to design the rule. However, PDApp keeps the classification performance on these new data.

## 4. Discussion

In dentistry, impacted lower third molars (M3s) can lead to complications if left untreated or surgical extraction is delayed, highlighting the significance of early detection and appropriate treatment planning. Dentists typically utilize panoramic radiography to identify the presence of M3s and perform radiological follow-ups of their progression, underscoring the importance of accurate detection. The preoperative prediction of third molar eruption with artificial intelligence (AI) has been a challenge in dentistry [5,6]. For example, a recent study [17] proposes an Explainable Mandibular Third Molar Convolutional Neural Network (E-mTMCNN) architecture, which incorporates Transfer Learning (TL), image preprocessing methods, and Local Interpretable Model-Agnostic Explanations (LIME), an explainable artificial intelligence (XAI) approach, for detecting the presence of mandibular M3s. Initially, a novel m-TM dataset was created for this problem and made publicly available, and various image preprocessing methods, such as Gaussian filtering, gamma correction, and data augmentation, were applied to enhance the performance of the proposed method on this dataset. Afterward, these preprocessed datasets were trained and tested with the CNN architectures employed in the TL method. Among these, the mTMCNN architecture based on GoogLeNet demonstrated the highest performance on the preprocessed m-TM dataset, achieving 87.02% accuracy, 75% sensitivity, 94.73% specificity, 77.68% precision, a 75.51% F1-score, and 87.01% AUC. Additionally, XAI results were obtained using LIME on test PRs, demonstrating the proposed method’s robust decision-making capability regarding the presence of mandibular M3s.

Decision support system software was developed using the proposed method and used in an expert survey prepared for doctor evaluation. The survey results indicated positive feedback from expert dentists, supporting the method’s performance. According to the performance comparison with the state-of-the-art methods presented in the literature, the PDApp revealed a superior performance regarding the performance metrics used. This software is a computer-aided diagnostic tool for the early diagnosis of the M3 eruption status, complications related to M3s in dentistry, and appropriate planning.

PDApp is a computer-aided diagnostic tool for the early diagnosis of M3 eruption status, complications related to M3s in dentistry, and appropriate planning. According to the performance comparison with the state-of-the-art methods presented in the literature, the PDApp revealed a superior performance to discriminate between retained and erupted third molar. The value of the PDApp is that it can be easily and quickly used on panoramic images in clinical practice. Results on the dataset used in the current study suggest that the RRC feature alone can predict the eruption status. However, further analysis on additional data revealed that it is not robust enough to account for the variability among cases, and Cos erupθ is further required for an accurate prediction. In conclusion, the universal behavior of the PDApp has been demonstrated, which is not influenced by the patient’s race or sex or whether the patient may or may not need an orthodontic procedure.

The value of the PDApp is that it can be easily and quickly used on panoramic images in clinical practice. Throughout this study, it was demonstrated, using the Kolmogorov–Smirnov test, that the RRC distribution in the erupted-M3 group, group E (whether or not an orthodontic procedure was performed), is different from that in the retained-M3 groups. This implies that this variable (the RRC) is of interest for classifying molars into these groups, as its behavior depends on the group. This result is reinforced by the ROC curves associated with the RRC, which have an associated AUC equal to 1 (the maximum possible value), demonstrating the excellent discriminatory power of this variable. In other words, the RRC is the most effective and efficient variable for distinguishing between erupted and retained molars. There is no evidence that the behavior of the Cos erupθ in the retained-molar groups is different from that in the erupted-molar group, in contrast to the RRC. In fact, the analysis of the ROC curve associated with the Cos erupθ to compare erupted and retained molars that have not undergone an orthodontic procedure has an associated AUC of 0.612, very close to 0.50, which represents a classification based solely on chance. In summary, the high discriminatory power of the RRC stands out compared with the almost negligible discriminative ability of the Cos erupθ. Therefore, for the classification of impacted and erupted M3s, the good performance of the PDApp is justified by the positive results associated with the RRC. Furthermore, good results were obtained both in patients who had not undergone orthodontic procedures and in those who had. The analyses conducted were not satisfactory in the case of the Cos erupθ, and thus, it would make sense to stop using this variable for the classification of erupted and retained M3s using the PDApp. In other words, the data collection required to use the PDApp could be simplified while maintaining its good results. In conclusion, the universal behavior of the PDApp has been demonstrated, which is not influenced by the patient’s race or sex or whether the patient may or may not need an orthodontic procedure.

## 5. Conclusions and Future Work

In this study, we confirmed the reliability of the PDApp for identifying the third molar eruption potential (erupted or retained status) in panoramic radiological images, achieving a final accuracy of up to 100%. The PDApp is a reliable and easy-to-use software tool for the estimation of the third molar eruption potential from the panoramic radiological images of adolescent patients. This contribution has the potential to improve the effectiveness and efficiency of dental image analysis and the rapid management of impacted mandibular third molars because the PDApp reduces the under-/overestimation problem, which is fairly common in manual and visual methods.

Nevertheless, certain limitations remain, and future work will incorporate the automatic calculation of the RRC on panoramic radiographs to build a fully automatic tool for eruption diagnosis prediction.

## Figures and Tables

**Table 1:** Main characteristic measures associated with retention coefficient and cosine of eruption angle in RSO, E, and RCO groups.

	RSO Group		E Group		RCO Group	
	RRC	Cos erupθ	RRC	Cos erupθ	RRC	Cos erupθ
Minimum	0.1620	−0.9998	0.7313	−0.9937	0.1352	−0.9998
First quartile	0.3114	−0.8961	1.0944	−0.4626	0.2234	−0.6070
Median	0.3845	−0.3133	1.2835	0.0691	0.3032	0.0979
Average	0.3939	−0.1858	1.3319	0.0659	0.3251	0.0705
Third quartile	0.4806	0.4202	1.5576	0.6669	0.4157	0.8592
Maximum	0.7135	0.9897	2.2047	0.9971	0.7109	0.9985

**Table 2:** *p*-values associated with Kolmogorov–Smirnov-type tests for pairwise comparative analysis of RRC.

	RSO Group	RCO Group	E Group
RSO Group	-	0.0081	<2.2 × 10^−16^
RCO Group	0.0081	-	<2.2 × 10^−16^
E Group	<2.2 × 10^−16^	<2.2 × 10^−16^	-

**Table 3:** Confusion matrix for rule-based system on the dataset used to train the PDApp. The row and column identify true and predicted class labels, respectively.

True/Predicted	Retained	Erupted
**Retained**	23	92
**Erupted**	0	73

## Data Availability

The data presented in this study are available upon request from the corresponding author. The data are not publicly available due to privacy reasons.
